# Will the COVID-19 infection affect the performance of top basketball players? A data-driven analysis

**DOI:** 10.3389/fspor.2024.1440472

**Published:** 2024-11-12

**Authors:** Changyue Xiong, Chenxi Wu, Lu Bai, Yuxin Yan, Sumeng Chen

**Affiliations:** Department of Sports Medicine, Peking University Shenzhen Hospital, Shenzhen, Guangdong, China

**Keywords:** COVID-19, elite basketball players, performance, return to play, National Basketball Association

## Abstract

**Purpose:**

To investigate the changes in the game performance of high-level basketball players in NBA league before and after the COVID-19 infection.

**Methods:**

Athletic statistics were collected from official database for 68 players (19 forwards, 29 guards, 20 centers; mean age 27.14 ± 3.65 years) in the NBA league for 10 games before and after infection with COVID-19. We used a the Kruskal-Wallis analysis of variance to examine the impact caused by COVID-19.

**Results:**

Among all 68 players, a statistically significant decrease in plus/minus (*Z* = −2.392, *p* = 0.017) and free throw shooting (FT% *Z* = −2.153, *p* = 0.031), occurred compared to the pre-infection with COVID-19. Among the interior players, we found a statistically significant decrease in free throw shooting FT% (*Z* = 2.674, *p* = 0.021), Plus/Minus (*Z* = −2.371, *p* = 0.018). Among centers and forwards, there was no statistically significant change.

**Conclusion:**

The impact of COVID-19 on players’ fatigue and cardiorespiratory and muscular endurance may have contributed to the decline in performance, and the impact of detraining due to isolation should not be ignored.

## Introduction

Since it was first detected in December 2019, COVID-19, which is caused by SARS-CoV-2 infection, has spread widely across the world. It has caused hundreds of millions of infections and millions of deaths. In 2020, in response to the COVID-19 outbreak, major sports leagues in North America and Europe chose to suspend play and then gradually resume play as the outbreak calmed down. National Basketball Association (the NBA league) announced a lockout in March 2020 after the first case of infection. From July through October, the NBA reopened the match in Orlando at a closed campus known as the “Bubble”. While the rematch in the Bubble was a success in terms of immunization (1), many players became infected with SARS-CoV-2 after the gradual return to regular play from the Bubble, and the impact of COVID-19 infection on player health and performance is a matter of debate.

The main symptoms of COVID-19 reported so far are mainly: fever, cough, shortness of breath in addition to chills, myalgia, headache and fatigue, which are also more frequently seen (2). Although the prognosis of COVID-19 is generally considered favorable, there has been a recent increase in the number of reports of residual symptoms of COVID-19 infection (post-COVID-19 syndrome) or long COVID. Residual symptoms of COVID-19 infection have been found in 32.6% to 87% of hospitalized patients (3). Fatigue, headache, dyspnea, and anosmia are more common in LONG COVID (4). Professional athletes who considered better physical condition than general population. As for athletes who are in better physical condition than the general population, some studies have suggested that in patients with COVID-19 infection, the better the patient's physical condition and cardiorespiratory fitness, the less severe the symptoms of COVID-19 infection are (5). Another study found that in seasons affected by the COVID-19 epidemic, the incidence of injuries among NBA players was not higher than in the two seasons prior to the COVID-19 epidemic (6).

Some studies have also shown that players infected with COVID-19 prior to the Bubble season did not experience a significant decrease in performance during the Bubble season (7). However, the Bubble season lasted more than four months from suspension to reinstatement, and the current NBA suspension for COVID-19 has been limited to individuals, and the duration of the suspension has been dramatically shortened, there has not been much research done to determine the effects of COVID-19 on player athleticism and player performance after returning to the game in this situation.

Basketball competition needs high intensity and longtime physical confrontation, requires excellent physical fitness of the players in order to perform well. Therefore, a decline in athleticism will affect the player's on-court performance, such as losing rebounds and being broken by opposing players, which will eventually be reflected in the game statistics. Previous research has shown that athletes who recover from Achilles tendon tears experience significant decreases in player efficiency rating (PER) and other statistics (8–10). However, there are no studies on the effects of COVID-19 on players’ game performance. This study used publicly available open-source information to collect game data from elite basketball players before and after they were infected with COVID-19 and attempted to analyze the changes in game data in an attempt to illustrate the effects of infection with COVID-19 on players’ on-court performance and athletic ability. The results of the study will increase awareness of COVID-19 and will be helpful in the development of disease prevention programs for leagues and teams.

## Method

### Player selection

Inclusion criteria: (1) NBA League Players; (2) announced by the official NBA website or official team/player social media to confirm the diagnosis of COVID-19 infection; (3) Appeared in an NBA regular season game (excluding playoffs) within three months before and after diagnosis; (4) Appeared in more than 3 games both before and after COVID-19 infections; Exclusion criteria: (1) suffered a major injury that resulted in a season-ending injury; (2) a player who experienced a trade.

A total of 68 players, all male with a mean age of 26.60 ± 4.02 years, were finally included in this study. Among them: 19 forward players with a mean age of 26.32 ± 4.40 years; 29 guard players with a mean age of 27.14 ± 3.65 years; and 20 centers with a mean age of 26.10 ± 4.25 years.

As the data for this study were collected from open data and the personal information of the players does not appear in the article, this article did not require specific approval from the ethics committee for this study.

### Data acquisition

Source of information on COVID-19 infected players were collected in NBA League Official Website. Official social media for each player and team. Specialized sports media: www.espn.com. Data of player performance were recorded from NBA League Official Website: https://www.nba.com/stats/players. Our statistics include demographic data: age, position, Time to return to the game and the player's game stats: points (PTS), Field Goals Made (FGM), Field Goals Attempted (FGA), Field Goals Percentage (FG%), 3 Point Field Goals Made (3PM), 3 Point Field Goals Attempted 3PA, 3 Point Field Goals Percentage (3P%), Free Throws Made (FTM), Free Throws Attempted (FTA), Free Throws Percentage (FT%), Offensive Rebounds (OREB), Defensive Rebounds (DREB), Rebounds (REB), Assists (AST), Stell (STL), Blocks (BLK), Turnovers (TOV), Personal Fouls (PF), Plus/Minus (+/−), Minutes Played (MIN). Integration data such as PER cannot be used as it requires comparisons based on overall league performance.

### Analysis and processing of data

With current players usually playing more than one position in the game, players were categorized into three groups of guards (G), Forwards (F), and center (C) according to the position on the court where they appeared the most and their style of play. Comparisons of player data before and after infection of COVID-19 were made within all players and within each group of centers, forwards, and guards. Since the COVID-19 infections among NBA players were reported by the official website, randomization in player selection was not possible. Therefore, we use non-parametric tests to compare the difference between groups.

We compared all 20 items of data collected in the forward, guard, and center groups, respectively. Separately: points (PTS), Field Goals Made (FGM), Field Goals Attempted (FGA), Field Goals Percentage (FG%), 3 Point Field Goals Made (3PM), 3 Point Field Goals Attempted 3PA, 3 Point Field Goals Percentage (3P%), Free Throws Made (FTM), Free Throws Attempted (FTA), Free Throws Percentage (FT%), Offensive Rebounds (OREB), Defensive Rebounds (DREB), Rebounds (REB), Assists (AST), Stell (STL), Blocks (BLK), Turnovers (TOV), Personal Fouls (PF), Plus/Minus (+/-), Minutes Played (MIN).

We first examined the relationship between COVID-19 infection and players’ age in our study: we found that the mean age of players infected with COVID-19 was 26.60 ± 4.02 years. The average age of forward players was 26.32 ± 4.40 years; the average age of guard players was 27.14 ± 3.65 years; and the average age of centers was 26.10 ± 4.25 years (Table 1). And according to publicly available data, in 2022, the average player age in the NBA league will be 26.1 years old (11, 12), which is essentially the same as our statistics on the average age of players infected with COVID-19. We used a non-parametric Kruskal-Wallis analysis of variance to test for differences in age between groups and calculated (*p* = 0.307). Therefore, we concluded that age was not an influential factor in players’ infection with COVID-19.

**Table 1:** The average age and average length of absence of the players.

	Centers	Forward	Guards	All players
Age	26.10 ± 4.25	26.32 ± 4.40	27.14 ± 3.65	26.60 ± 4.02
Average length of absence	56.05 ± 59.51	40.47 ± 56.86	60.3 ± 62.75	53.53 ± 53.53

We also counted the length of time players missed with injuries due to COIVD-19 infection, and the average length of absence for all players was 54 days. However, because the NBA experienced a lockout at the time of the initial outbreak in 2020, this lockout resulted in a generally longer average absence time for players infected during the lockout. In 2020, the average injury absence was 141.1 ± 31.9 days; in 2021, the average injury absence was 17.2 ± 6.0 days; and in 2022, the average injury absence was 16.3 ± 17.8 days. Thus except for 2020, which received the effects of the suspension, the time for a player to return from a COVID-19 infection was approximately 2 weeks.

## Results

### Changes in performance of all players after COVID-19 infection

**Figure 1:**
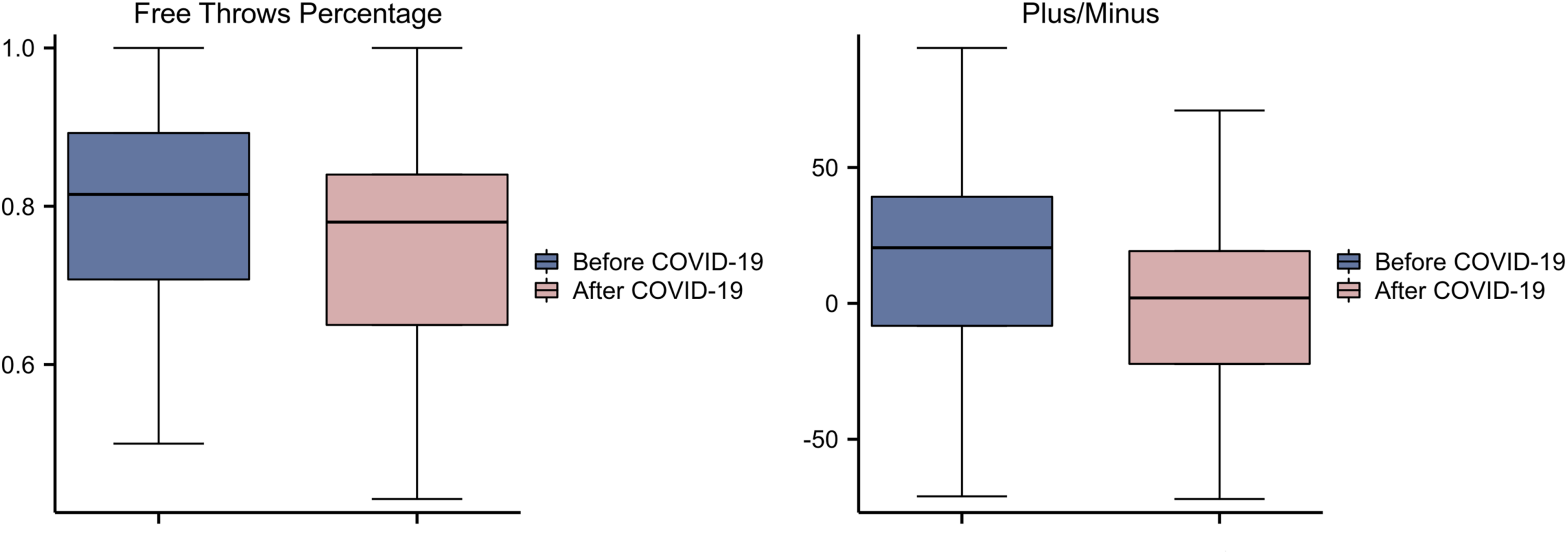
Free throws percentage, plus/minus of all players before and after COVID-19 infection.

Across all 68 players, we found statistically significant changes in plus/minus and FT%: Average FT% before COVID-19: 0.8 ± 0.14, Average FT% after COVID-19: 0.74 ± 0.19, *Z* = −2.153, *p* = 0.031. Average plus/minus before COVID-19: 15.78 ± 40.93, average plus/minus after COVID-19: 2.29 ± 38.41, *Z* = −2.392, *p* = 0.017. To avoid redundancy, we give all the data where statistically significant changes occur in a Figure 1; and all the statistical data were shown in Table 2.

**Table 2:** Performance metrics of all players before and after COVID-19 infection.

	Before COVID-19	After COVID-19	*Z*	*p*
PTS	12.6 ± 6.88	12.47 ± 7.41	−0.263	0.793
FGM	4.55 ± 2.66	4.6 ± 2.56	−0.35	0.726
FGA	9.59 ± 5.2	9.74 ± 5.38	−0.469	0.639
FG%	0.48 ± 0.11	0.46 ± 0.13	−1.564	0.118
3PM	1.32 ± 1.04	1.25 ± 0.93	−1.027	0.305
3PA	3.55 ± 2.46	3.57 ± 2.32	−0.055	0.956
3P%	0.32 ± 0.16	0.29 ± 0.16	−1.464	0.143
FTM	2.06 ± 1.58	2.11 ± 1.94	−0.222	0.824
FTA	2.57 ± 1.98	2.71 ± 2.36	−0.742	0.458
FT%*	0.8 ± 0.14	0.74 ± 0.19	−2.153	0.031
OREB	1.13 ± 0.94	1.12 ± 0.91	−0.057	0.954
DREB	4.07 ± 1.98	4.05 ± 2.33	−0.866	0.387
REB	5.21 ± 2.61	5.17 ± 2.86	−0.528	0.598
AST	2.9 ± 2.42	2.64 ± 2.3	−1.907	0.056
STL	0.79 ± 0.51	0.75 ± 0.43	−0.572	0.568
BLK	0.63 ± 0.6	0.55 ± 0.49	−1.202	0.229
TOV	1.41 ± 1.11	1.47 ± 1.02	−1.263	0.207
PF	2.05 ± 0.69	2.09 ± 0.8	−0.866	0.387
+/−*	15.78 ± 40.93	2.29 ± 38.41	−2.392	0.017
MIN	25.27 ± 7.91	24.83 ± 8.75	−0.764	0.445

* *p* < 0.05.

### Changes in performance of centers after COVID-19 infection

**Figure 2:**
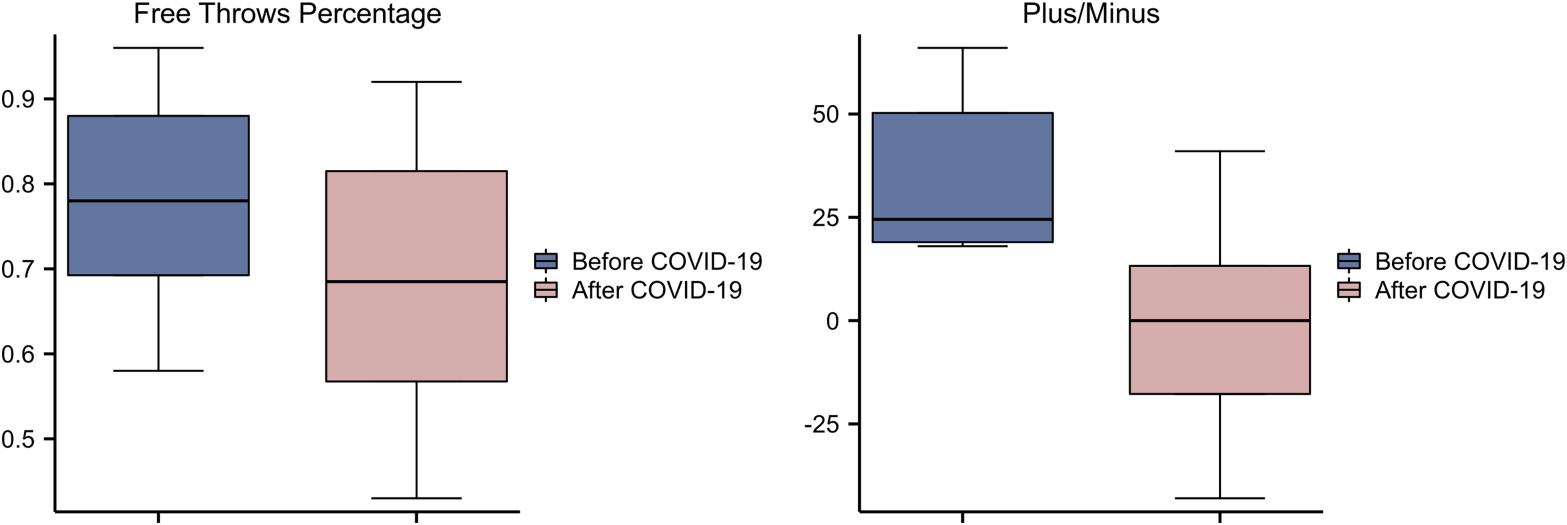
Free throws percentage, plus/minus of centers before and after COVID-19 infection.

Among the centers, we found a statistically significant change in plus/minus and FT% (Figure 2, Table 3).

**Table 3:** Performance metrics of centers before and after COVID-19 infection.

	Before COVID-19	After COVID-19	*Z*	*p*
PTS	12.04 ± 5.95	12.78 ± 7.06	−1.027	0.305
FGM	4.87 ± 2.5	4.43 ± 1.99	−1.176	0.239
FGA	8.29 ± 4.55	9.12 ± 5.13	−1.195	0.232
FG%	0.57 ± 0.12	0.54 ± 0.15	−0.896	0.37
3PM	0.72 ± 0.8	0.81 ± 0.95	−0.550	0.582
3PA	1.95 ± 2.03	2.19 ± 2.36	−0.285	0.776
3P%	0.23 ± 0.23	0.22 ± 0.22	−0.454	0.65
FTM	2.47 ± 1.92	2.24 ± 1.99	−1.107	0.268
FTA	3.09 ± 2.22	3.04 ± 2.39	−0.588	0.556
FT%*	0.77 ± 0.14	0.66 ± 0.22	−2.315	0.021
OREB	1.92 ± 1	1.85 ± 1.03	−0.181	0.856
DREB	5.24 ± 2.06	4.9 ± 2.51	−1.248	0.212
REB	7.16 ± 2.56	6.76 ± 3.08	−0.896	0.37
AST	1.95 ± 1.62	1.82 ± 1.78	−0.503	0.615
STL	0.65 ± 0.38	0.7 ± 0.32	−0.458	0.647
BLK	1.13 ± 0.73	0.99 ± 0.54	−1.089	0.276
TOV	1.38 ± 0.9	1.43 ± 0.84	−0.181	0.856
PF	2.27 ± 0.61	2.35 ± 0.81	−0.897	0.37
+/−*	28.05 ± 39.08	7.35 ± 35.6	−2.371	0.018
MIN	22.71 ± 7.16	22.65 ± 8.05	−0.560	0.575

* *p* < 0.05.

Average FT% before COVID-19: 0.77 ± 0.14, Average FT% after COVID-19: 0.66 ± 0.22, *Z* = 2.674, *p* = 0.021. Average plus/minus before COVID-19: 28.05 ± 39.08, average plus/minus after COVID-19: 7.35 ± 35.6, *Z* = −2.371, *p* = 0.018.

### Changes in performance of forwards after COVID-19 infection

Among the forwards, there was no statistically significant change (Table 4).

**Table 4:** Performance metrics of forwards before and after COVID-19 infection.

	Before COVID-19	After COVID-19	*Z*	*p*
PTS	12.56 ± 8.08	12.28 ± 9.3	−0.684	0.494
FGM	4.52 ± 3.37	4.7 ± 3.09	−0.866	0.387
FGA	9.57 ± 5.75	9.93 ± 6.28	−0.719	0.472
FG%	0.46 ± 0.1	0.41 ± 0.14	−1.69	0.091
3PM	1.36 ± 0.97	1.14 ± 0.73	−1.809	0.07
3PA	3.69 ± 2.25	3.59 ± 1.94	−0.302	0.763
3P%	0.33 ± 0.14	0.29 ± 0.13	−1.677	0.094
FTM	1.79 ± 1.55	2.11 ± 2.33	−0.61	0.542
FTA	2.35 ± 2.21	2.7 ± 2.93	−0.483	0.629
FT%	0.76 ± 0.15	0.77 ± 0.11	−0.233	0.816
OREB	1.04 ± 0.84	1.16 ± 0.77	−1.113	0.266
DREB	4.1 ± 2.06	4.45 ± 2.51	−0.564	0.573
REB	5.14 ± 2.54	5.61 ± 2.74	−0.784	0.433
AST	2.5 ± 1.87	2.18 ± 2.03	−1.530	0.126
STL	0.7 ± 0.47	0.67 ± 0.45	−0.109	0.913
BLK	0.59 ± 0.47	0.46 ± 0.42	−1.321	0.187
TOV	1.18 ± 1.07	1.32 ± 1.04	−1.572	0.116
PF	1.82 ± 0.76	1.79 ± 0.83	−0.161	0.872
+/−	1.47 ± 39.09	−3 ± 37.91	−0.342	0.732
MIN	26.37 ± 9.76	25.91 ± 10.90	−0.282	0.778

### Changes in performance of guards after COVID-19 infection

Among guards, there was no statistically significant change (Table 5).

**Table 5:** Performance metrics of guards before and after COVID-19 infection.

	Before COVID-19	After COVID-19	*Z*	*p*
PTS	13.01 ± 6.86	12.38 ± 6.46	−0.735	0.462
FGM	4.36 ± 2.3	4.66 ± 2.61	−0.854	0.393
FGA	10.49 ± 5.22	10.04 ± 5.07	−0.854	0.393
FG%	0.44 ± 0.07	0.43 ± 0.06	−0.205	0.837
3PM	1.72 ± 1.07	1.64 ± 0.91	−0.552	0.581
3PA	4.56 ± 2.36	4.51 ± 2.1	−0.125	0.9
3P%	0.37 ± 0.09	0.35 ± 0.08	−0.524	0.6
FTM	1.96 ± 1.31	2.02 ± 1.69	−0.125	0.9
FTA	2.35 ± 1.63	2.5 ± 1.96	−0.524	0.6
FT%	0.84 ± 0.12	0.77 ± 0.19	−1.389	0.165
OREB	0.66 ± 0.55	0.59 ± 0.46	−0.876	0.381
DREB	3.24 ± 1.46	3.2 ± 1.81	−0.584	0.559
REB	3.9 ± 1.8	3.79 ± 2.09	−0.779	0.436
AST	3.81 ± 2.89	3.51 ± 2.54	−1.128	0.259
STL	0.96 ± 0.57	0.84 ± 0.46	−1.078	0.281
BLK	0.31 ± 0.25	0.32 ± 0.24	−0.201	0.841
TOV	1.57 ± 1.26	1.6 ± 1.13	−0.541	0.588
PF	2.05 ± 0.68	2.1 ± 0.73	−0.467	0.64
+/−	16.69 ± 41.75	2.28 ± 41.35	−1.406	0.16
MIN	26.31 ± 6.86	25.62 ± 7.61	−0.984	0.325

## Discussion

When athletes return to sports from the recovery of a COVID-19 infection, they will face challenges on multiple conditions: Not only physical, but also mental and psychological challenges. To cope with the high intensity of professional basketball, fatigue and muscle soreness are the first challenges that arise. Gattoni et al. noted that fatigue was the most predominant symptom experienced by athletes with COVID-19 infection (13), while another study of hospitalized patients noted that 70% of patients experienced fatigue after discharge (14). furthermore, the respiratory system condition. With COVID-19-induced coughing and the potential to exacerbate an athlete's pre-existing respiratory condition being another factor that may affect an athlete's return to play (15–17). Another possible cause that may affect an athlete's performance is the effect of detraining. Detraining refers to the partial or complete loss of functional adaptations to physical activity resulting from the cessation of training. causes a decrease in strength, speed, flexibility and endurance by affecting cardiorespiratory and neuromuscular function (18, 19). A study of Basketball players in Europe league found that a decline in player stats after the league restarted (20), which the authors attribute to the effects of psychological stress, physical, technical ability, and unfavorable schedules. However, the study included all players involved in the tournament and therefore could not specify the effect of COVID-19 on the players.

Since previous studies have shown that most COVID-19-induced symptoms can gradually subside within 1 month (4, 17).our study focused on changes in performance in short period of time before and after infection with COVID-19 as a research theme. Among all players, we found statistically significant changes in plus/minus and FT%. Positive and negative values refer to the number of points a player scores/loses on the court, with higher positive values resulting in more team wins and lower negative values resulting in more team losses. We found that the positive-negative value decreased from 15.78 ± 40.93 to 2.29 ± 38.41 after the COVID-19 infected player returned to the court. this indicates that the player's ability to help the team decreased. In addition, the player's PTS, FG%, 3P%, FT%, REB, AST, STL, and BLK all declined, while other negative data, such as turnovers and fouls, increased instead. We therefore conclude that although some of these do not appear to be statistically significant changes in the data, there has been a (small) decline in overall performance since the players returned to play.

One reason for this may be due to the effects of COVID-19 on cardiorespiratory functions in athletes. Good cardiorespiratory fitness is key to keeping athletes playing well throughout the game, even though basketball is not an endurance sport (21). And female runners discharged after COVID-19 infection were found to have decreased cardiorespiratory endurance and elevated self-fatigue scores in a study of female runners (22). Some patients with long COVID, on the other hand, showed decreased performance on the 6-minute walk test (23). Another study in a younger population showed a 10% decrease in VO2 max in patients infected with COVID-19 (24), so it is possible that decreased cardiorespiratory endurance and fatigue due to COVID-19 infections may be responsible for the players’ decreased performance.

We also observed that the free throw percentage of the players after infection with COIVD-19 declined, and the free throw percentage of the inside players declined more than that of the other players. It is known that for the free throw shooting rate, it is generally lower for inside players than for guards and forwards; also, the higher the intensity of the game, the lower the free throw shooting percentage, for example, the free throw shooting percentage in the playoffs is lower than that in the regular season (25). In our study, the decrease in free throw shooting percentage was greater for inside players than for other players. This may be because the effects of COVID-19 make players less tolerant to high-intensity play. Previous studies have noted that fatigue affects centers more than guards (26). In another study comparing the difference in endurance between centers and guards, there was no difference between the players in the first two sprint tests, but in the third consecutive middle sprint, guards were faster than centers (27); since the centers need to cover, assist, and scramble for rebounds frequently during the game. As a result, centers require more physical confrontation than guards and forwards, and are more physically demanding than forwards and guards. Therefore, the impact of COVID-19 on player fatigue and endurance is more likely to be seen in the interior than in other positions and is reflected in free throw shooting percentages. Inside players also showed a greater decrease in positive and negative values than guards and forwards. From 28.05 ± 39.08 to 7.35 ± 35.6, *Z* = −2.371, *p* = 0.018; in contrast, the *Z* values for forwards and guards in this category were −0.342 and −1.406, and did not show a significant difference.

Another reason for a player's declining stats could be detraining. Detraining is very common among athletes, and the end of the season, injuries, and illnesses can all be reasons that cause detraining. Isolations and suspensions due to COVID-19 have been shown to have a detrimental effect on an athlete's return to competition (28, 29). And even a 2-week Detraining can lead to a decline in an athlete's sprint performance (28). In our study, the average absence of players was 54 days, enough to cause a decline in athleticism and lead to a decline in player statistics.

## Conclusions

To conclude, among all players, the overall performance of the players has decreased by the impact of COVID-19, in which all the stats have decreased, and the most obvious decreases are in +/− plus/minus and FT% Free Throw Shooting Percentage. Among the players in each group, centers saw a greater decline in their stats, which we believe is due to the fact that centers play a more physical style of play and are therefore more affected by COVID-19. We believe that the mechanism by which COVID-19 affects player performance may be due to the effect on player fatigue with cardiorespiratory and muscular endurance; in addition, detraining due to isolation is also a contributing factor to the decline in player performance.

There were some limitations in this study: first, since the players are all suspended after the infection, the players’ performance may also be affected by the suspension. Second, the data we collected is not a complete season, in which the players’ opponents are different, teammates are different, and the coaches’ use of the players may also be different, which may also have an impact on the players’ data. Third, plus/minus is a kind of cumulative data, will also receive the impact of playing time, not very suitable for direct comparison of the performance of the player good or bad. Fourth, this study only examined the statistics of the NBA league and it would be more meaningful to compare the performance of infected players in the major basketball leagues around the world, and we will implement this plan in future studies.

## Data Availability

The raw data supporting the conclusions of this article will be made available by the authors, without undue reservation.
